# The predictability of serum anti-Müllerian level in IVF/ICSI outcomes for patients of advanced reproductive age

**DOI:** 10.1186/1477-7827-9-115

**Published:** 2011-08-15

**Authors:** Robert KK Lee, Frank SY Wu, Ming-Huei Lin, Shyr-Yeu Lin, Yuh-Ming Hwu

**Affiliations:** 1 Department of Obstetrics and Gynecology, Mackay Memorial Hospital, Taipei, Taiwan; 2 Department of Obstetrics and Gynecology, Taipei Medical University, Taipei, Taiwan; 3 Mackay Medicine, Nursing and Management College, Taipei, Taiwan

## Abstract

**Background:**

The role of serum anti-Müllerian hormone (AMH) as predictor of in-vitro fertilization outcomes has been much debated. The aim of the present study is to investigate the practicability of combining serum AMH level with biological age as a simple screening method for counseling IVF candidates of advanced reproductive age with potential poor outcomes prior to treatment initiation.

**Methods:**

A total of 1,538 reference patients and 116 infertile patients aged greater than or equal to 40 years enrolled in IVF/ICSI cycles were recruited in this retrospective analysis. A reference chart of the age-related distribution of serum AMH level for Asian population was first created. IVF/ICSI patients aged greater than or equal to 40 years were then divided into three groups according to the low, middle and high tertiles the serum AMH tertiles derived from the reference population of matching age. The cycle outcomes were analyzed and compared among each individual group.

**Results:**

For reference subjects aged greater than or equal to 40 years, the serum AMH of the low, middle and high tertiles were equal or lesser than 0.48, 0.49-1.22 and equal or greater than 1.23 ng/mL respectively. IVF/ICSI patients aged greater than or equal to 40 years with AMH levels in the low tertile had the highest cycle cancellation rate (47.6%) with zero clinical pregnancy. The nadir AMH level that has achieved live birth was 0.56 ng/mL, which was equivalent to the 36.4th percentile of AMH level from the age-matched reference group. The optimum cut-off levels of AMH for the prediction of nonpregnancy and cycle cancellation were 1.05 and 0.68 ng/mL, respectively.

**Conclusions:**

Two criteria: (1) age greater than or equal to 40 years and (2) serum AMH level in the lowest tertile (equal or lesser than 33.3rd percentile) of the matching age group, may be used as markers of futility for counseling IVF/ICSI candidates.

## Background

One of the most difficult aspects of assisted reproductive treatment (ART) is to identify and counsel patients with very low or virtually no chance of achieving live birth. A report by the Ethics Committee of the American Society of Reproductive Medicine (ASRM) nevertheless has provided a valuable guideline when considering fertility treatment for patients with extremely poor prognosis. According to the report, patients are defined as having "poor prognosis" when the likelihood of a given ART treatment achieving live birth is very low but not nonexistent (>1% and ≤ 5% per cycle); while those with essentially no chance of achieving live birth with ART treatment (≤ 1% per cycle) are considered as "futility" [1].

Prior to the advancements of ultrasound and immunoassay, the initial assessment of fecundity was largely based on one's chronological age. Although the age-related decline in reproductive capacity has been well documented in general population [2], the rate of fertility decline can vary considerably among individual women of the same age [3,4], indicating that "ovarian aging" may not be unequivocally dictated by chronological aging. With the paradigms of modern ART emphatically stressing the importance of treatment individualization and optimization, other markers are clearly in need.

Lacking a precise definition, the ovarian age is best represented by ovarian reserve, the functional potential of ovaries at any given time. Before initiating ART treatment, ovarian reserve can be assessed ultrasonically by antral follicle count (AFC), the ovarian volume and ovarian blood flow, by endocrine markers such as basal follicular stimulating hormone (FSH), inhibin-B, estradiol and anti-Müllerian hormone (AMH), and/or by ovarian stimulatory tests such as the clomiphene citrate challenge test (CCCT), the exogenous FSH ovarian reserve test (EFORT) and the gonadotropin agonist stimulation test (GAST) [3]. The ultimate goal of these tests is to provide a more accurate prediction of the potential success for patients prior to treatment initiation, thus enabling a more feasible, patient oriented treatment approach.

The aim of this study is therefore to assess the practicability of using serum AMH level, in conjunction with chronological age, for screening and counseling advanced-aged IVF candidates with high probability of poor outcomes prior to treatment initiation. For the present study, advanced age is defined as equal or above forty years old, and poor outcomes are defined as IVF/ICSI cycles resulting either in cancellation or nonpregnancy.

## Methods

### Study subjects

Medical records were retrospectively examined for patients who sought fertility consultation or treatment at the Infertility Division of the Department of Obstetrics-Gynecology, Mackay Memorial Hospital from December 1st, 2006 to May 31st, 2010. The study protocol was approved by the Institutional Review Board of Mackay Memorial Hospital in Taipei, Taiwan.

For the reference population, the inclusion criteria were patients who fitted the medical definition of infertility "one year of unprotected intercourse but not pregnant" and without any of the following exclusion criteria: (1) menopause or premature ovarian failure status (Day 3 serum FSH level > 10 IU/mL), (2) history of ovarian or adnexal surgery, (3) suspicious findings of ovarian malignancy, and (4) presence of endocrine disorders such as diabetes mellitus, hyperprolactinemia, thyroid dysfunction, congenital adrenal hyperplasia, Cushing's syndrome, adrenal insufficiency, and polycystic ovary syndrome. In addition, severe underweight or overweight patients were also excluded (BMI < 20 or > 27). For the analysis of IVF patients ≥ 40 years of age, the inclusion criteria were the same as the ones for reference population, with the addition of having undergone IVF during the same period of time.

### Study design

The variation of AMH levels among different races and ethnicities has been well documented in the study by Seifer et al. [5]. Since the chart for age-related distribution of AMH levels has not been established for Asian population, it was pertinent that an age-specific reference values must first be formulated. After applying the above exclusion criteria, AMH data of 1,538 infertility patients between the ages of 23 to 46 were obtained. For each age group, the AMH levels were further stratified into respective percentiles, serving as reference values for the next phase of this study.

The relationship between age and serum AMH levels on IVF/ICSI outcomes in infertility patients ≥ 40 years of age was investigated in the following phase. Of all the reference infertility patients ≥ 40 years of age (n = 378), only the ones who had undergone IVF/ICSI were enrolled (n = 116). These 116 subjects were further divided into three groups according to the low, middle and high tertiles (≤33.3^rd ^percentile, 33.4-66.6^th ^percentile, and ≥ 66.7^th ^percentile) from the AMH reference values for the 378 patients ≥ 40 years of age. The cycle outcomes were then analyzed and compared among each individual group.

The nadir serum AMH levels capable of achieving live birth were identified and ranked according to the reference AMH values of corresponding reference age groups. Correlations between AMH levels and outcomes were also observed for the infertility patients aged 35 or younger who underwent IVF/ICSI during the same period (n = 323). Lastly, the serum AMH cut-off values for the prediction of nonpregnancy and cycle cancellation for IVF/ICSI patients with aged 40 and above were further quantified using the receiver operating characteristic (ROC) curve analysis.

### Ovarian stimulation protocols

All IVF/ICSI patients received either the GnRH agonist long protocol or the GnRH antagonist protocol. All patients received a starting dosage of 375 IU of recombinant follicle-stimulating hormone (r-FSH; Gonal-F; Serono Laboratories, Aubonne, Switzerland) and 150 IU of human menopausal gonadotropin (hMG; Menopur; Ferring GmbH, Kiel, Germany) for three consecutive days, and the dosage was then adjusted every two to three days in accordance with the follicle growth until the day of hCG administration. For the GnRH agonist group, leuprolide acetate (Takeda Pharma GmbH, Stolberg, Germany) was given at a daily dose of 1 mg, starting on the twenty-first day of the previous menstrual cycle until the serum levels of estradiol fell below 30 pg/mL, and patients then began daily gonadotropin injection. In the GnRH antagonist group, patients began daily administration of r-FSH and hMG on the second or third day of the menstrual cycle. After at least one follicle reached 14 mm in diameter, daily injection of 0.25 mg of cetrorelix (Cetrotide; Serono, Baxter Oncology GmbH, Halle, Germany) was given until the day of hCG administration.

When at least two follicles reached 18 mm in diameter, 10,000 IU hCG (Pregnyl; Schering-Plough, Kenilworth, NJ, USA) was administered and oocyte retrieval was performed 34-36 hours later. Conventional IVF or ICSI was conducted 4-6 hours post oocyte retrieval. For IVF, each oocyte was inseminated with 20 × 10^3 ^motile spermatozoa in a single droplet containing 20 μl of fertilization medium (Quinn's Advantage Fertilization medium; SAGE IVF Inc. Trumbull, Connecticut, U.S.A.). For ICSI, 1-2 μl washed spermatozoa were placed in 7% polyvinylpyrrolidone (PVP; SAGE IVF Inc.) and a sperm was injected into each denuded oocyte using standardized techniques. Each embryo was cultured in a single droplet containing 20 μl of medium (Quinn's Advantage Cleavage medium; SAGE IVF Inc.) and incubated under the atmospheric composition of 5% CO_2_, 5% O_2 _and 90% N_2 _at 37°C. All embryo transfers were performed at 72 hours post oocyte retrieval.

### Luteal support and confirmation of pregnancy

The luteal phase was supported by intramuscular injection of 50 mg of progesterone and vaginal supplementation of 300 mg micronized progesterone (Progeffik; Effik, Paris, France) or Crinone 8% progesterone gel (Columbia Laboratories, Inc., Livingston, NJ) once per day. Serum hCG was measured 14 days after oocyte retrieval and a value above 5 IU/ml was designated as positive pregnancy. Clinical pregnancy was defined as a pregnancy diagnosed by ultrasonographic visualization of the gestational sac. Viable pregnancy was defined as gestation age greater than 7^th ^weeks with documented fetal cardiac activity by ultrasound.

### Serum AMH measurement

AMH levels were measured by enzyme-linked immunosorbent assay kit (ELISA, Diagnostic Systems Laboratories, Webster, TX). The detection range of the assay was between 0.025-15 ng/mL with the detection limit (0.017 ng/mL). Values below the detection limits were considered as zero. The intra-and-interassay variation coefficients were 4.6% and 8.0% respectively. Samples from all subjects were obtained via venipuncture and analyzed by the same laboratory (Department of Immunoassay, Mackay Memorial Hospital, Taipei, Taiwan). The samples were processed according to the manufacturer instructions by centrifuging at 1400 × g for 10 minutes to separate cellular contents and the debris, then the serum was transferred to sterile polypropylene tubes to be preserved at -70°C until assayed.

### Statistical analysis

Statistical analysis was performed using SigmaPlot (Systat Software Inc., Chicago, IL, USA) and MedCalc 10.2 (MedCalc Software, Mariakerke, Belgium). Continuous variables were presented as mean and standard deviation (SD). For categorical variables, the values were presented as raw frequencies with corresponding percentages, and the between-group differences were assessed either by Chi square test with Yates correction if needed, or by Fisher exact test. The 95% confidence interval of the binomial distribution for clinical pregnancies in each of the three groups were calculated by the modified Wald method. Receiver operating characteristic (ROC) curves were generated to investigate the predictability of AMH levels in IVF cycle cancellation and nonpregnancy. The sensitivity, specificity, positive and negative predictive values were calculated for the optimal AMH cut-off levels determined by ROC curve analysis. The results were considered statistically significant at p < 0.05.

## Results

Both the mean and median AMH values of the reference group showed a progressive decline with advancing age (Table 1). The low, middle, and high tertiles of serum AMH for reference subjects with aged 40 years and above were ≤0.48, 0.49-1.22 and ≥1.23 ng/mL respectively. The mean age differences among the three groups analyzed by ANOVA were found to be statistically insignificant. The sample size was considered sufficient by one tailed post hoc power analysis (α = 0.05 and a power of 80%).

**Table 1:** The age-related distribution chart of serum AMH level (ng/mL) for reference infertility patients

Age	Mean ± SD	5^th ^centile	10^th ^centile	25^th ^centile	Median	75^th ^centile	90^th ^centile	95^th ^centile	n
**≤**	4.07 ± 1.98	1.74	1.88	2.61	3.38	5.46	7.12	8.57	36
**27**	3.82 ± 1.93	0.30	1.39	2.45	3.43	5.17	6.42	7.60	30
**28**	4.41 ± 2.01	0.77	1.80	2.78	4.47	5.75	7.33	7.70	45
**29**	3.79 ± 1.09	1.08	1.41	2.27	3.48	5.31	6.74	7.16	70
**30**	3.77 ± 1.96	1.03	1.15	2.26	3.38	5.52	6.57	6.90	82
**31**	3.61 ± 2.14	0.83	1.23	1.81	3.20	4.84	6.85	7.68	104
**32**	3.80 ± 2.08	0.68	1.11	2.23	3.62	5.21	6.97	7.68	127
**33**	3.29 ± 1.90	0.88	1.09	1.66	2.91	4.63	5.97	6.80	129
**34**	2.81 ± 1.99	0.53	0.75	1.24	2.35	3.78	6.01	7.18	149
**35**	3.15 ± 1.89	0.67	0.83	1.70	2.83	4.51	5.79	6.57	104
**36**	3.08 ± 1.83	0.81	1.06	1.33	2.77	4.38	5.40	6.57	86
**37**	2.61 ± 1.65	0.75	0.78	1.12	2.47	3.49	5.12	5.67	69
**38**	2.03 ± 1.40	0.06	0.19	0.88	1.91	2.94	3.87	4.64	73
**39**	1.45 ± 1.35	0.05	0.11	0.48	1.07	2.02	3.14	4.65	56
**40**	1.68 ± 1.36	0.11	0.13	0.63	1.42	2.41	3.65	4.20	126
**41**	1.18 ± 1.32	0.02	0.05	0.29	0.86	1.35	3.04	3.48	88
**42**	0.91 ± 0.97	0.01	0.05	0.23	0.60	1.24	2.34	3.53	47
**43**	0.79 ± 0.77	0.00	0.05	0.06	0.63	1.50	2.09	2.48	43
**44**	0.69 ± 0.61	0.06	0.08	0.28	0.52	0.98	1.36	2.36	32
**45**	0.66 ± 0.66	0.13	0.14	0.32	0.54	0.67	1.12	2.73	26
**46**	0.50 ± 0.40	0.00	0.00	0.10	0.52	0.81	1.11	1.14	16
**All**	2.71 ± 2.04	0.14	0.40	0.65	2.36	3.09	5.78	6.82	1538
**≥ 40**	1.17 ± 1.99	0.05	0.06	0.32	0.78	1.56	2.91	3.53	378

The IVF/ICSI outcomes for subjects of age ≥ 40 years are presented in Table 2. After dividing them according to the low, middle and high tertiles from the AMH reference values for patients with age ≥ 40 years, a trend of negative cycle outcomes was observed toward the lower end of AMH tertile. Subjects with AMH concentrations in the low tertile had zero clinical pregnancy, which differed significantly from those of the middle (23.7%, p < 0.02) and the high tertiles (29.8%, p < 0.0001). On the other hand, women with AMH concentrations in the low tertile had the highest rate of cycle cancellation (47.6%), which also differed significantly from those of the middle (15.7%, p < 0.04) and the high tertiles (1.7%, p < 0.01). When comparing the outcomes between the higher two tertiles, a significant difference was noted only for cycle cancellation (15.7% vs. 1.7%, p = 0.02) but not for clinical pregnancy rate (23.7% vs. 29.8%, p > 0.05). The 95% confidence interval of the binomial distribution for clinical pregnancies from the low to high tertile was zero to 16 percent, 13-39 percent and 19-42 percent respectively.

**Table 2:** Outcomes of IVF/ICSI cycles in women of advanced reproductive age (≥ 40 years old)

		Serum AMH tertiles (ng/mL)		
	Total subjects	Low (≤ 0.48)	Middle (0.49~1.22)	High (≥ 1.23)
**Total cycle**	116	21	38	57
**Cancelled cycle (%)**	17 (14.7)	10 (47.6)^§^	6 (15.7)*	1 (1.7)
**Mean age**	41.5 ± 1.4	42.8 ± 2.3	41.1 ± 1.3	41.3 ± 1.4
**Peak E2 level (pg/mL)**	1542.1 ± 1146.5	802.6 ± 748.9	1050 ± 699	2095.8 ± 1156.2
**Number of oocytes retrieved**	6.1 ± 4.6	2.4 ± 2.9	4.7 ± 2.5	8.1 ± 5.1
**Number of mature oocytes**	4.7 ± 3.7	1.5 ± 1.73	3.9 ± 2.5	6.3 ± 3.9
**Embryos obtained**	3.8 ± 2.9	1.8 ± 2.4	3.1 ± 2.2	4.8 ± 3.1
**Embryos transferred**	2.8 ± 1.2	1.6 ± 1.3	2.6 ± 1.0	3.2 ± 1.0
**Clinical pregnancy per cycle (%)**	26 (22.4)	0 (0)^§^	9 (23.7)	17 (29.8)
**Abortion per cycle (%)**	7 (26.9)	0 (0)	0 (0)	7 (41.2)
**Viable pregnancy per cycle (%) [FHB (+) & > 7 wks.]**	19 (16.4)	0 (0)﹟	9 (23.7)	10 (17.5)

^§ ^p < 0.05 compared with the middle tertile and the high tertile

*p < 0.05 compared with the high tertile

﹟p < 0.05 compared with the middle tertile

The lowest serum AMH level that achieved live birth in the group aged ≥ 40 years was 0.56 ng/mL, which was equivalent to the 36.4^th ^percentile of AMH level from the reference group of the same age. For the group aged ≤35 years, live birth was achievable even for subjects with diminished ovarian reserve. The nadir AMH level that achieved live birth in that group was 0.26 ng/mL, which was equivalent to the 1.7^th ^percentile of AMH level from the reference group of the same age.

With respect to the prediction of cycle cancellation and nonpregnancy for infertility patients aged ≥ 40 years, AMH demonstrated a high discriminative ability with respective areas under curve (AUC) of 0.77 and 0.66 by ROC curve analysis. The optimal cut-off level of AMH for the prediction of IVF cycle cancellation was 0.68 ng/mL (p < 0.05), with specificity of 85.1% and negative predictive value of 92.0%. The optimal cut-off level of AMH for the prediction of IVF nonpregnancy was 1.05 ng/mL (p < 0.05), with specificity of 86.7% and positive predictive value of 91.4%. The cut-off levels of AMH for IVF cycle cancellation (0.68 ng/mL) and nonpregnancy (1.05 ng/mL) corresponded to the 43.8^th ^and 56.8^th ^percentile of the reference serum AMH level from the matching age group respectively. The complete sensitivity, specificity, positive and negative predictive values are summarized in Table 3.

**Table 3:** Prediction of cycle cancellation and nonpregnancy by serum AMH levels in women of advanced reproductive age (≥ 40 years old)

	Cut-off AMH level (ng/mL)	ROC_AUC_	Sensitivity (%)	Specificity (%)	PPV (%)	NPV (%)	Significance level
**Cycle cancellation**	0.68	0.77	64.7	85.1	47.8	92.0	p = 0.0001
**Nonpregnancy**	1.05	0.65	42.7	86.9	91.4	31.7	p = 0.022

## Discussion

The most pertinent concern to the infertile couple has always been the success rate of specific ART treatments. Therefore, numerous studies have been dedicated to identify reliable markers that may help in predicting outcomes in IVF cycles, as well as the feasibility of applying these markers in patient counseling prior to treatment initiation. A recent meta-analysis has concluded that female age, duration of subfertility, basal FSH level and number of retrieved oocytes are all predictors of pregnancy in IVF cycles [6]. However, many other potential markers, including serum AMH level, were not incorporated as the factors analyzed in that study.

Since its discovery, AMH has been a promising marker in various clinical setting of ART. Initially viewed as an accurate marker of ovarian reserve [7,8], AMH was subsequently found to be a reliable predictor of controlled ovarian hyperstimulation for both poor [9,10] and hyper responses [11,12]. In addition, one study reported AMH to be a competent surrogate marker for antral follicle count in the diagnosis of PCOS by the Rotterdam Criteria [13]. Nevertheless, the relationship between serum AMH level and IVF pregnancy outcome still has not been fully elucidated. A recent review by Broekmans et al. has concluded that ovarian reserve tests, including AMH, were not good predictors of pregnancy in IVF cycles [14].

There are several possible rationales for the poor performance of AMH in predicting pregnancy in IVF cycles. First of all, the international assay standard for AMH measurement is lacking. Up to dates, only few studies have attempted to create age-specific serum AMH values for the infertile population [15] and healthy cohorts [16,17]. Secondly, while these studies have all observed a similar trend of steady decrement of AMH level with advanced age, a wide range of distribution of AMH levels among each age group was also noted [15-17]. The wide variability was also observed in the AMH data from our reference group study, and that was one of the primary reasons that median was used for analysis instead of arithmetic mean. Furthermore, among most studies that investigated the predictability of AMH levels in IVF pregnancy, no specific age group was targeted for the enrolled subjects. Therefore, a wide range of cut-off values was presented (0.10-2.70 ng/mL) [7,18-25] with various sensitivity, specificity, positive and negative predictive values. In short, serum AMH value alone may not be a sufficient predictor of pregnancy in IVF cycles without other considering factors.

Furthermore, the quality and quantity of oocytes are both important determinants for the success in ART. Several studies have demonstrated that serum AMH may also possess the additional ability to predict the quality of oocytes and embryos [18,26-28], while others have failed to replicate such relationship [21,29,30]. In a well-designed study by Wang et al., the authors revealed that both the clinical pregnancy rate per retrieval and live birth rate per embryo transfer did not differ significantly across all three AMH tertiles (≤0.29, 0.30-1.20 and ≥1.21 ng/ml) for women aged <34 years. This indicated that favorable outcomes may still be attained for the infertile patients of younger age on the basis of biologically competent oocytes, despite of the diminished ovarian reserve [31]. The observation drawn from the present study also demonstrated that pregnancy was still achievable even in patients younger than 35 years old with extremely low AMH level (0.26 ng/mL; 1.7^th ^percentile). These findings concurred with the conclusions drawn from the recent meta-analysis by van Loendersloot et al., which found that female age, hence oocyte quality, was the most important predictor of pregnancy in IVF cycles among almost all of the analyzed studies [6].

On the other hand, the present study disclosed that for women of advanced reproductive age (≥ 40), having AMH concentrations in the higher two tertiles was associated with nonsignificant difference in clinical pregnancy rate, which differed significantly from those with AMH level in the lowest tertile. In addition, no live birth was achieved for women with AMH in the lowest tertile with extremely high cycle cancellation rate. These results demonstrated that for women ≥ 40 years old with AMH level in the lowest tertile, poor IVF/ICSI cycle outcomes could be expected due to poor oocyte quantity as well as quality. Though the range of AMH tertiles were defined differently, similar observation was also made in the study by Wang et al for women of advanced reproductive age [31].

One of the limitations of the present study was the relative small sample size (n = 116) in the IVF group ≥ 40 years old. Being a retrospective study which targeted only on a specific group of infertile population, a larger sample size is indeed needed in the future to confirm the results of this study. Furthermore, the results from the present study were to simply present an easier and more reliable counseling tool for evaluating and identifying patients of potential poor outcome, rather than a tool of exclusion. The patients should always have final decision of whether or not to receive treatment.

## Conclusions

To the best of our knowledge, this is the first ever study that investigated the lower limits of serum AMH level capable of achieving viable pregnancy in advanced-aged IVF/ICSI patients, specifically those equal and over forty years old. In addition, from our data, we were able to derive the serum AMH cut-off values that may reliably predict the likelihood of cycle cancellation as well as nonpregnancy for IVF/ICSI patients equal and over forty years old. To sum up, we propose two simple criteria for patient counseling: (1) age equal and over forty years and (2) serum AMH level in the low tertile (≤33.3^rd ^percentile) of the matching age group, as the markers of futility for IVF/ICSI due to high cancellation rate and slim chance for clinical pregnancy.

## Competing interests

The authors declare that they have no competing interests.

## Authors' contributions

RKKL participated in the original design of the study and drafted the manuscript. FSYW carried out the data collection and performed the statistical analysis. YMH supervised the study and validated the data. MHL and SYL assisted in the study organization and the data collection. All of authors have read and approved the final manuscript.

## References

[B1] The Ethics Committee of the American Society for Reproductive Medicine Fertility treatment when the prognosis is very poor or futile Fertil Steril 2009 92 1194 1197 19726040 10.1016/j.fertnstert.2009.07.979

[B2] Templeton A Morris JK Parslow W Factors that affect outcome of in-vitro fertilisation treatment Lancet 1996 348 1402 1406 10.1016/S0140-6736(96)05291-9 8937279

[B3] Broekmans FJ Kwee J Hendriks DJ Mol BW Lambalk CB A systematic review of tests predicting ovarian reserve and IVF outcome Hum Reprod Update 2006 12 685 718 10.1093/humupd/dml034 16891297

[B4] te Velde ER Pearson PL The variability of female reproductive ageing Hum Reprod Update 2002 8 141 154 10.1093/humupd/8.2.141 12099629

[B5] Seifer DB Golub ET Lambert-Messerlian G Benning L Anastos K Watts DH Cohen MH Karim R Young MA Minkoff H Greenblatt RM Variations in serum mullerian inhibiting substance between white, black, and Hispanic women Fertil Steril 2009 92 1674 1678 10.1016/j.fertnstert.2008.08.110 18930217 PMC3037722

[B6] van Loendersloot LL van Wely M Limpens J Bossuyt PM Repping S van der Veen F Predictive factors in in vitro fertilization (IVF): a systematic review and meta-analysis Hum Reprod Update 2010 16 577 589 10.1093/humupd/dmq015 20581128

[B7] Van Rooij IA Broekmans FJ te Velde ER Fauser BC Bancsi LF Jong FH Themmen AP Serum anti-Müllerian hormone levels: a novel measure of ovarian reserve Hum Reprod 2002 17 3065 3071 10.1093/humrep/17.12.3065 12456604

[B8] Tremellen KP Kolo M Gilmore A Lekamge DN Antimullerian hormone as a marker of ovarian reserve Aust N Z J Obstet Gynaecol 2005 45 20 24 10.1111/j.1479-828X.2005.00332.x 15730360

[B9] La Marca A Giulini S Tirelli A Bertucci E Marsella T Xella S Volpe A Anti-Müllerian hormone measurement on any day of the menstrual cycle strongly predicts ovarian response in assisted reproductive technology Hum Reprod 2007 22 766 771 17071823 10.1093/humrep/del421

[B10] Jayaprakasan K Campbell B Hopkisson J Johnson I Raine-Fenning N A prospective, comparative analysis of anti-Müllerian hormone, inhibin-B, and three-dimensional ultrasound determinants of ovarian reserve in the prediction of poor response to controlled ovarian stimulation Fertil Steril 2010 93 855 864 10.1016/j.fertnstert.2008.10.042 19046583

[B11] Lee TH Liu CH Huang CC Wu YL Shih YT Ho HN Yang YS Lee MS Serum anti-Müllerian hormone and estradiol levels as predictors of ovarian hyperstimulation syndrome in assisted reproduction technology cycles Hum Reprod 2008 23 160 167 18000172 10.1093/humrep/dem254

[B12] Nardo LG Gelbaya TA Wilkinson H Roberts SA Yates A Pemberton P Laing I Circulating basal anti-Müllerian hormone levels as predictor of ovarian response in women undergoing ovarian stimulation for in vitro fertilization Fertil Steril 2009 92 1586 1593 10.1016/j.fertnstert.2008.08.127 18930213

[B13] Pigny P Jonard S Robert Y Dewailly D Serum anti-Müllerian hormone as a surrogate for antral follicle count for definition of the polycystic ovary syndrome J Clin Endocrinol Metab 2006 91 941 945 16368745 10.1210/jc.2005-2076

[B14] Broer SL Mol B Dólleman M Fauser BC Broekmans FJ The role of anti-Müllerian hormone assessment in assisted reproductive technology outcome Curr Opin Obstet Gynecol 2010 22 193 201 10.1097/GCO.0b013e3283384911 20407372

[B15] Seifer DB Baker VL Leader B Age-specific serum anti-Müllerian hormone values for 17,120 women presenting to fertility centers within the United States Fertil Steril 2011 95 747 750 10.1016/j.fertnstert.2010.10.011 21074758

[B16] Shebl O Ebner T Sir A Schreier-Lechner E Mayer RB Tews G Sommergruber M Age-related distribution of basal serum AMH level in women of reproductive age and a presumably healthy cohort Fertil Steril 2011 95 832 834 10.1016/j.fertnstert.2010.09.012 20970126

[B17] La Marca A Sighinolfi G Giulini S Traglia M Argento C Sala C Masciullo C Volpe A Toniolo D Normal serum concentrations of anti-Müllerian hormone in women with regular menstrual cycles Reprod Biomed Online 2010 21 463 469 10.1016/j.rbmo.2010.05.009 20797903

[B18] Ebner T Sommergruber M Moser M Shebl O Schreier-Lechner E Tews G Basal level of anti-Müllerian hormone is associated with oocyte quality in stimulated cycles Hum Reprod 2006 21 2022 2026 10.1093/humrep/del127 16679324

[B19] Penarrubia J Fabregues F Manau D Creus M Casals G Casamitjana R Carmona F Vanrell JA Balasch J Basal and stimulation day 5 anti-Müllerian hormone serum concentrations as predictors of ovarian response and pregnancy in assisted reproductive technology cycles stimulated with gonadotropin- releasing hormone agonist-gonadotropin treatment Hum Reprod 2005 20 915 922 15665015 10.1093/humrep/deh718

[B20] Kwee J Schats R McDonnell J Themmen A de Jong F Lambalk C Evaluation of AMH as test for the prediction of ovarian reserve Fertil Steril 2008 90 737 743 10.1016/j.fertnstert.2007.07.1293 17923131

[B21] Smeenk JM Sweep FC Zielhuis GA Kremer JA Thomas CM Braat DD Antimüllerian hormone predicts ovarian responsiveness, but not embryo quality or pregnancy, after in vitro fertilization or intracyoplasmic sperm injection Fertil Steril 2007 87 223 226 10.1016/j.fertnstert.2006.06.019 17081531

[B22] Gnoth C Schuring AN Friol K Tigges J Mallmann P Godehardt E Relevance of anti-Müllerian hormone measurement in a routine IVF program Hum Reprod 2008 23 1359 1365 10.1093/humrep/den108 18387961

[B23] Elgindy EA El-Haieg DO El-Sebaey A Anti-Müllerian hormone: correlation of early follicular, ovulatory and midluteal levels with ovarian response and cycle outcome in intracytoplasmic sperm injection patients Fertil Steril 2008 89 1670 1676 10.1016/j.fertnstert.2007.05.040 17658520

[B24] Barad DH Weghofer A Gleicher N Comparing anti-Müllerian hormone (AMH) and follicle-stimulating hormone (FSH) as predictors of ovarian function Fertil Steril 2009 91 1553 1555 10.1016/j.fertnstert.2008.09.069 19217095

[B25] Eldar-Geva T Ben Chetrit A Spitz IM Rabinowitz R Markowitz E Mimoni T Gal M Zylber-Haran E Margalioth EJ Dynamic assays of inhibin B, anti-Müllerian hormone and estradiol following FSH stimulation and ovarian ultrasonography as predictors of IVF outcome Hum Reprod 2005 20 3178 3183 10.1093/humrep/dei203 16113044

[B26] Hazout A Bouchard P Seifer DB Aussage P Junca AM Cohen-Bacrie P Serum antimüllerian hormone/müllerian-inhibiting substance appears to be a more discriminatory marker of assisted reproductive technology outcome than follicle-stimulating hormone, inhibin B, or estradiol Fertil Steril 2004 82 1323 1329 10.1016/j.fertnstert.2004.03.061 15533354

[B27] Silberstein T MacLaughlin DT Shai I Trimarchi JR Lambert-Messerlian G Seifer DB Keefe DL Blazar AS Müllerian inhibiting substance levels at the time of HCG administration in IVF cycles predict both ovarian reserve and embryo morphology Hum Reprod 2006 21 159 163 16123085 10.1093/humrep/dei270

[B28] Lekamge DN Barry M Kolo M Lane M Gilchrist RB Tremellen KP Anti-Müllerian hormone as a predictor of IVF outcome Reprod Biomed Online 2007 14 602 610 10.1016/S1472-6483(10)61053-X 17509203

[B29] Lie Fong S Baart EB Martini E Schipper I Visser JA Themmen AP de Jong FH Fauser BJ Laven JS Anti-Müllerian hormone: a marker for oocyte quantity, oocyte quality and embryo quality? Reprod Biomed Online 2008 16 664 670 10.1016/S1472-6483(10)60480-4 18492370

[B30] La Marca A Sighinolfi G Radi D Argento C Baraldi E Artenisio AC Stabile G Volpe A Anti-Müllerian hormone (AMH) as a predictive marker in assisted reproductive technology (ART) Hum Reprod Update 2010 16 113 130 10.1093/humupd/dmp036 19793843

[B31] Wang JG Douglas NC Nakhuda GS Choi JM Park SJ Thornton MH Guarnaccia MM Sauer MV The association between anti-Müllerian hormone and IVF pregnancy outcomes is influenced by age Reprod Biomed Online 2010 21 757 761 10.1016/j.rbmo.2010.06.041 21044868

